# Updates in Management of SARS-CoV-2 Infection

**DOI:** 10.3390/jcm11154472

**Published:** 2022-07-31

**Authors:** Robert Flisiak, Dorota Zarębska-Michaluk, Marta Flisiak-Jackiewicz, Piotr Rzymski

**Affiliations:** 1 Department of Infectious Diseases and Hepatology, Medical University of Białystok, 15-540 Białystok, Poland; 2 Department of Infectious Diseases, Jan Kochanowski University, 25-317 Kielce, Poland; dorota1010@tlen.pl; 3 Department of Pediatrics, Gastroenterology, Hepatology, Nutrition and Allergology, Medical University of Białystok, 15-247 Białystok, Poland; m_flisiak@op.pl; 4 Department of Environmental Medicine, Poznan University of Medical Sciences, 60-806 Poznań, Poland; rzymskipiotr@ump.edu.pl

## 1. Introduction

Severe acute respiratory syndrome coronavirus 2 (SARS-CoV-2) has spread worldwide since the beginning of 2020 [1]. Its infections are mostly asymptomatic or mild, but some patients may develop COVID-19 (coronavirus disease 2019) with a severe or critical course leading to pneumonia, acute respiratory distress syndrome, and multi-organ failure. Apart from the virus-related damage to the lungs, pathomechanism of the disease seems to be linked to thromboembolism and inflammation accompanied by overproduction of proinflammatory cytokines [2]. Since developing new therapeutic molecules, dedicated strictly to targeting a particular virus is time-consuming [3], scientists and physicians have started to test and repurpose old medications in clinical practice [4]. Despite the introduction of antiviral drugs and immunomodulators, after two and a half years of pandemics, there is still a lack of optimal therapy. A major issue is also insufficient knowledge on predictors of the severe or deadly course of the disease, which could also help to switch from one therapeutic option to another. Due to many gaps in the management of COVID-19, there is a need for accumulating new data, particularly from real-world experience which could be applicable to practice guidelines. The objective of this Special Issue of the Journal of Clinical Medicine was to provide an update on the management for the diagnostic workup and therapy of SARS-CoV-2 infection. The issue includes fourteen original articles covering problems related to the diagnosis, clinic, and treatment of COVID-19, with an emphasis on predictors of severity of the disease.

## 2. Clinical Picture

The study based on the SARSTer database analyzed the data of 5199 COVID-19 patients hospitalized in 30 Polish centers in periods of dominance of various SARS-CoV-2 variants [5]. It showed some shifts in SARS-CoV-2 pathogenicity between March 2020 and July 2021 in the Polish cohort of hospitalized patients. A share of patients presenting respiratory, systemic, and gastrointestinal symptoms was higher in the later phase of a pandemic than in the first three months. Interestingly there was no shift in the age of admitted patients and patients who died throughout the studied period. No gender difference in fatality rate was seen, although the age of males who died was significantly lower. It is also plausible that other factors had influenced the shift in disease severity and outcome throughout the considered period as a separate analysis of the SARSTer database has shown the relationship between patients’ exposure to increased levels of air pollution and inflammation, need for oxygen therapy, and odds of death due to COVID-19 [6]. Data in the pediatric population from the initial period of the pandemic are presented in the study by Pokorska-Śpiewak et al. [7]. The authors showed that the characteristics of pediatric patients infected with SARS-CoV-2 and the clinical presentation of COVID-19 are age-related. Younger children were more frequently infected by close relatives, and they more often suffered from pneumonia and gastrointestinal symptoms and require hospitalization. The results of these two studies can be considered as a reference for further analyses conducted under the dominance of new SARS-CoV-2 variants [5,7].

Based on data from 455 patients with previously diagnosed kidney failure, Zarębska-Michaluk et al. [8] showed that this population was characterized by significantly older age and a more severe clinical course of COVID-19. The age, baseline SpO2, need for oxygen therapy, neutrophil and platelet count, estimated glomerular filtration rate, C-reactive protein concentration, and some comorbidities were the independent predictors of 28-day mortality in this population. This analysis clearly showed that underlying kidney disease in patients with COVID-19 should be considered one of the leading factors associated with a higher risk of severe clinical presentation and mortality.

Moniz et al. [9] presented a case series of five patients coinfected with cytomegalovirus (CMV) admitted to the intensive care unit due to respiratory failure related to COVID-19. The authors speculate that the reason for the reactivation was the immunosuppression possibly associated with COVID-19. However, they emphasize the importance of multiple confounding factors usually associated with immunosuppression, such as the clinical profile of older patients with multiple comorbidities, the critical illness itself, or immunosuppressive treatments should also be considered.

## 3. Diagnostics

The article by Seynaeve Y. et al. [10] published in June 2021 documents research on the usefulness of antigen tests in the diagnosis of COVID-19. It was a time of confusion related to the appearance of a large number of such tests, often of poor quality, which undermined the credibility of this diagnostic method. Thanks to such works, only antigen tests with diagnostic effectiveness similar to the RT-PCR technique remained in use, and the speed and convenience caused a real revolution in diagnostic and epidemiological procedures. Of course, the RT-PCR technique remains the standard for the diagnosis, but technical difficulties often require supporting the diagnosis with other methods. The work of Principe et al. [11] proved that the genetic test should be combined with pulmonary CT scans, clinical pictures, and some inflammatory blood tests, to increase the accuracy of the diagnosis of COVID-19.

Since the beginning of the COVID-19 pandemic, medical imaging has been assigned a key role in the diagnosis of the disease. However, a question arose if and to which extent automated tools could be included in clinical diagnosis. Artificial intelligence, has started to play an increasing role in medicine, including COVID-19. Jemioło et al. [12] investigated the methodological quality of the reviews on artificial intelligence techniques to diagnose COVID-19 in medical images. Unfortunately, the authors found that most of the reviews included less than 10% of available studies, which makes it difficult to collect and organize knowledge.

## 4. Predictors of the Outcome

A significant part of the publications included in the Special Issue were works devoted to the search for predictors of the clinical course of COVID-19. Tamayo-Velasco et al. [13] investigated the role of inflammatory cytokines using the 45-plex Human XL Cytokine Luminex Performance Panel and found that three of them may be predictive. High levels of hepatocyte growth factor and interleukin-1α accompanied with low levels of interleukin-27 at admission can predict clinical outcomes. Of course, the predictive value depends on the availability of the methodology in laboratories. Therefore, in another work, Ramos-Lopez et al. [14] focused on simple indicators of liver and proinflammatory features as determinants of COVID-19 morbidity and fatal outcomes. They found the predictive values of ROC curves for FIB-4, aminotransferases (AST/ALT) ratio, C-reactive protein, Charlson Comorbidity Index, neutrophils, and platelets concerning intensive care and death outcomes. In turn, Pastrovic et al. [15] showed that patients with more severe liver injury more frequently experienced higher rates of intensive care unit admission, mechanical ventilation, and mortality.

Due to the fact that SARS-CoV-2 uses angiotensin-converting enzyme 2 (ACE-2) as a receptor enabling human infection, research has been undertaken on a possible relationship between angiotensin 1 receptor (AT1R) levels in the serum and the course of the disease. However, Janc et al. [16] did not show the effect of AT1R on the severity of symptoms associated with COVID-19 among healthcare professionals and any prognostic significance. On the other hand, San-Cristobal et al. [17], based on the analysis of clinical and biochemical variables obtained in the first 72 h of hospitalization from 1039 COVID-19 patients, specified three clusters with different clinical severity outcomes. These clusters displayed mortality from below 2% (cluster A), through around 15% (B) to as much as 40% (C) in patients with multi-organ lesions and significantly altered inflammatory and immune responses.

## 5. Treatment

The pathogenesis of COVID-19 includes, in addition to direct viral effect and coagulopathy, an overproduction of proinflammatory cytokines termed a cytokine storm, which is responsible for organ damage and is considered a major reason for death due to COVID-19. Tocilizumab, an antagonist of the interleukine-6 receptor, has emerged as a promising therapeutic choice, especially for the severe form of the disease. A systematic review and meta-analysis by Maraolo et al. [18] confirmed this view, pointing to the need for further studies to consolidate these findings and to identify the populations that benefit most from treatment with tocilizumab. Such a population was indicated in another study published in this Special Issue. Tocilizumab was found to be a therapeutic option to significantly reduce mortality and speed up clinical improvement in patients with a baseline concentration of interleukin-6 over 100 pg/mL, particularly if they need oxygen supplementation due to SpO2 values below 90% [19].

## 6. Conclusions

This editorial highlights the key findings of the research published in this Special Issue of the Journal of Clinical Medicine. We strongly encourage you to read particular papers for a detailed understanding of the reported results. The articles were published between April 2021 and May 2022; therefore, reading them, you must be aware of how quickly our knowledge of COVID-19 has changed. Nevertheless, most of the information in these publications is still valid and influences the management of patients
